# ATF4 Activation Promotes All-*trans*-Retinal-Driven Retinopathies in Mice

**DOI:** 10.3390/antiox15101328

**Published:** 2026-10-14

**Authors:** Kunhuan Yang, Chaofeng Yu, Danxue He, Lei Tao, Bo Yang, Yuling Chen, Jingmeng Chen, Yalin Wu

**Affiliations:** 1 Xiamen Eye Center and Eye Institute of Xiamen University, School of Medicine, Xiamen University, Xiamen 361003, China; 2 Fujian Provincial Key Laboratory of Ophthalmology and Visual Science, Fujian Engineering and Research Center of Eye Regenerative Medicine, Eye Institute of Xiamen University, School of Medicine, Xiamen University, Xiamen 361102, China; 3 School of Medicine, Xiamen University, Xiamen 361102, China; 4 Shenzhen Research Institute of Xiamen University, Shenzhen 518057, China

**Keywords:** ATF4, GSDME, mitochondria, macular degeneration, Stargardt disease

## Abstract

Impaired clearance of all-*trans*-retinal (atRAL) during the visual cycle leads to retinal degeneration, which is closely associated with autosomal recessive Stargardt disease (STGD1) and dry age-related macular degeneration (dry AMD). Transient activation of activating transcription factor 4 (ATF4) generally supports cellular adaptation to stress, whereas excessive ATF4 expression or prolonged activation can become maladaptive and promote oxidative and metabolic disturbances. Here, we reported that activation of ATF4 promoted atRAL-induced photoreceptor damage through mitochondria-caspase/DNA damage-dependent apoptosis and gasdermin E (GSDME)-dependent pyroptosis and apoptosis, and identified ATF4 as an upstream transcriptional regulator of GSDME. Genetic ablation of *Atf4* or pharmacological attenuation of ATF4 signaling with the integrated stress response inhibitor ISRIB visibly preserved the viability and morphology of atRAL-treated 661W cone photoreceptor cells. Both interventions lowered GSDME N-terminal fragment (GSDME-N) abundance, decreased intracellular and mitochondrial reactive oxygen species (ROS) production, and blunted damage to mitochondria and DNA. Notably, pharmacologically suppressing ATF4 expression in the neural retina effectively prevented retinal function loss and structural injury as well as photoreceptor apoptosis and pyroptosis in light-exposed *Abca4^−/−^Rdh8^−/−^* mice, which exhibit severe defects in atRAL clearance and recapitulate key features of STGD1 and dry AMD. Our findings demonstrate that ATF4 is a critical mediator of atRAL-induced retinal atrophy, suggesting that ATF4 inhibition represents a promising therapeutic strategy for STGD1 and dry AMD.

## 1. Introduction

In the canonical visual cycle, photoisomerization of 11-*cis*-retinal to all-*trans*-retinal (atRAL) within photoreceptors is rapidly followed by its reduction to all-*trans*-retinol and subsequent recycling through the retinal pigment epithelium (RPE) [1,2]. This tightly regulated process depends on two key proteins, ATP-binding cassette subfamily A member 4 (ABCA4) and retinol dehydrogenase 8 (RDH8), which mediate efficient atRAL clearance and thereby prevent its excess accumulation [3]. Defective clearance of this aldehyde and the resulting retinal injury are central features of atrophic macular degeneration, mainly including autosomal recessive Stargardt disease (STGD1) and the dry form of age-related macular degeneration (dry AMD) [4,5,6]. Accumulating evidence indicates that *Abca4* is the pathogenic gene of STGD1, while both *Abca4* and *Rdh8* serve as risk genes for dry AMD [3,6,7]. As previously reported, mice lacking both *Abca4* and *Rdh8* (*Abca4^−/−^Rdh8^−/−^* mice) display significantly elevated atRAL levels in the retina and recapitulate key features of STGD1 and dry AMD, particularly progressive photoreceptor and RPE degeneration [4,8]. Light exposure accelerates the release of atRAL from rhodopsin, causing a rapid surge of this toxic metabolite in the retina, which in turn speeds up the atrophy of photoreceptors and the RPE [1,4,9,10]. Collectively, these findings establish atRAL toxicity as a key driver of retinal damage in atrophic macular degeneration.

The integrated stress response (ISR) functions as a highly conserved cellular surveillance mechanism that detects diverse environmental and pathological insults, including proteostasis defects, nutrient deprivation, and oxidative stress, and orchestrates the delicate balance between adaptive survival and maladaptive cellular outcomes [11]. Activating Transcription Factor 4 (ATF4) functions as a central effector of the ISR. Upon stress-dependent phosphorylation of eukaryotic initiation factor 2α (eIF2α), the translational machinery selectively permits ATF4 translation, thereby enabling rapid induction of a transcriptional program that regulates redox balance, autophagy, mitochondrial quality control, and cell death pathways [11,12].

During the early stage of the response, ATF4-mediated transcriptional activation facilitates adaptive recovery [13,14]. However, sustained ATF4 activation drives maladaptive remodeling characterized by metabolic dysregulation, chronic oxidative stress and engagement of pro-death programs [14,15,16,17]. Emerging evidence implicates aberrant ATF4 signaling in diverse pathological conditions including neurodegeneration and ocular diseases [18,19,20]. Importantly, genetic or pharmacological inhibition of ATF4 ameliorates diabetes-associated vascular retinopathy and attenuates neuronal cell loss [15,21,22,23]. Despite these insights, the mechanisms by which ATF4 contributes to the pathogenesis of dry AMD and STGD1 remain unclear. Gasdermin E (GSDME) serves as the key effector driving pyroptosis onset [24,25]. We have previously shown that atRAL promotes GSDME cleavage in 661W cone photoreceptor cells and in neural retinas of light-exposed *Abca4^−/−^Rdh8^−/−^* mice, whereas GSDME deficiency attenuates plasma membrane rupture, mitochondrial damage, and photoreceptor death [26]. While signal transducer and activator of transcription 3 (STAT3), p53, and methyltransferase-like 3 (METTL3)-mediated m6A modification have been implicated in GSDME regulation across other biological contexts [27,28,29], the transcriptional control mechanisms governing GSDME in photoreceptors remain undefined.

In this study, we employed complementary in vitro and in vivo models characterized by defective atRAL clearance to examine the role of ATF4 in mediating retinal degeneration associated with atRAL-related retinopathies such as STGD1 and dry AMD, and to evaluate its interplay with GSDME.

## 2. Materials and Methods

### 2.1. Reagents and Antibodies

A detailed list of reagents and antibodies is provided in Table S1 of the Supplementary Information.

### 2.2. Animals

*Abca4*^−/−^*Rdh8*^−/−^ mice on a C57BL/6J background (lacking the *Crb1-rd8* allele) were maintained and genotyped as described [9]. Animal protocols were approved by the Institutional Animal Care and Use Committee of Xiamen University School of Medicine and complied with the CCRVO guidelines. In light-damage experiments, four-week-old mice were dark-adapted for 48 h before i.p. injection of ISRIB (5 mg/kg) or DMSO. Two hours later, pupils were dilated with 1% tropicamide, and mice were exposed to 10,000 lx white light-emitting diode (LED) light for 1 h. Daily i.p. injections continued thereafter. Eyes were harvested on day 5. Control animals received ISRIB or vehicle without light exposure.

### 2.3. Cell Culture

Murine 661W cone photoreceptor cells (Shanghai Zishi Biotechnology, Shanghai, China) were cultured in Dulbecco’s modified Eagle’s medium (DMEM; Gibco, Suzhou, China) supplemented with 10% fetal bovine serum (FBS; HyClone, Logan, UT, USA) and 1% penicillin–streptomycin. Cell cultures were maintained at 37 °C in a humidified incubator with 5% CO_2_.

### 2.4. atRAL Treatment

Cell seeding density was adjusted according to the assay: 1 × 10^4^ cells/well for 96-well plates or 3 × 10^5^ cells/well for 6-well plates, with overnight adherence prior to treatment. atRAL was dissolved in dimethyl sulfoxide (DMSO) to generate a 40 mM stock, which was freshly diluted in serum-free DMEM to 5 μM before use. Cells were then exposed to atRAL for 3 h or 6 h as specified. Control groups received a matching volume of DMSO, maintaining a final solvent concentration of 0.0125% (*v*/*v*). Given the light-sensitive nature of atRAL, all handling steps were performed under subdued light.

### 2.5. Bioinformatic Analysis

RNA-sequencing datasets were retrieved from our previously published work [30], comprising three independent biological replicates per group. Differential expression analysis was conducted using DESeq2 based on raw gene-level read counts. Counts were normalized via DESeq2 size factors, and differential expression between atRAL-treated and control cells was evaluated using a negative binomial generalized linear model with the Wald test. *p* values were adjusted using the Benjamini–Hochberg procedure, and genes meeting the criteria of adjusted *p* < 0.05 and |log_2_ fold change| ≥ 1 were designated as differentially expressed. All downstream analyses and visualizations were carried out in R (version 4.4.3). Volcano plots were produced using ggplot2 and ggrepel. Kyoto Encyclopedia of Genes and Genomes (KEGG) over-representation analysis was executed using clusterProfiler (version 4.14.6) and org.Mm.eg.db (version 3.20.0), with the full set of genes tested in the differential expression analysis and successfully mapped to Entrez identifiers serving as the background universe. Pre-ranked gene set enrichment analysis (GSEA) was performed using clusterProfiler and fgsea (version 1.32.4), and positive normalized enrichment scores (NESs) indicated enrichment toward the atRAL-treated group. For focused heatmap visualization, sample-level FPKM (Fragments Per Kilobase of transcript per Million mapped reads) values were transformed as log_2_(FPKM + 1), standardized to gene-wise Z scores across samples, and displayed using pheatmap (version 1.0.12). A predefined, literature-curated ISR–ATF4-associated gene panel was similarly visualized using pheatmap. Genes and samples were presented in predefined orders without hierarchical clustering.

### 2.6. ISRIB Treatment

661W cells were pretreated with ISRIB (2 or 4 μM) for 2 h. The pretreatment medium was then removed and replaced with fresh serum-free DMEM containing ISRIB at the corresponding concentration (2 or 4 μM) together with atRAL (5 μM). After an additional 6 h of incubation, the cells were collected for subsequent analyses. The corresponding control groups underwent the same medium-replacement procedure and received equivalent volumes of DMSO.

### 2.7. Generation of Atf4^−/−^ 661W Cell Line

A target sequence in exon 3 of mouse *Atf4* (CTATGTAAAGCGTCTCCACG) was identified using the CRISPR design tool from the Massachusetts Institute of Technology (https://crispor.gi.ucsc.edu) (accessed on 13 June 2022). Guide RNA (gRNA) oligonucleotides (see Supplementary Table S2) were annealed into duplexes and inserted into the *BsmBI* site of the pL-CRISPR.EFS.GFP vector (Addgene, Watertown, MA, USA). Resulting plasmids were transfected into 661W cells with Lipofectamine™ LTX & PLUS™ reagent (Thermo Fisher Scientific, Waltham, MA, USA) according to the manufacturer’s instructions. At 24 h post-transfection, GFP-positive single cells were sorted by a MoFlo Astrios flow cytometer (Beckman Coulter Life Sciences, Indianapolis, IN, USA) into 96-well plates and cultured for 14 days. Knockout clones were confirmed by Western blotting. Wild-type (WT) and *Atf4*^−/−^ 661W cells were exposed to 5 μM atRAL for 6 h. The *Gsdme*^−/−^ 661W cell line was previously established via CRISPR/Cas9 [26].

### 2.8. Cell Viability Assay

After the designated treatments, the medium in 96-well plates was aspirated and replaced with 100 μL of fresh complete medium containing 20 μL of CellTiter 96^®^ AQueous One Solution reagent (MTS; Promega, Madison, WI, USA). Following a 1 h incubation under standard culture conditions, absorbance was measured at 490 nm using a microplate reader (Thermo Fisher Scientific, Waltham, MA, USA).

### 2.9. Lactate Dehydrogenase (LDH) Release Assay

Extracellular LDH release was measured with an LDH cytotoxicity assay kit (Cat. No. C0016; Beyotime, Shanghai, China) as described previously [26]. Percentage release was calculated as (sample release–medium background)/(maximum release–medium background) × 100. The maximum-release reference was prepared by complete lysis of control cells with the lysis reagent supplied in the kit; cell-free wells containing the same volume of culture medium were used as background controls.

### 2.10. Detection of Reactive Oxygen Species (ROS)

WT and *Atf4^−/−^* 661W cells were cultured in 6-well plates and treated with 5 μM atRAL or DMSO for 6 h. Cells were then loaded with 10 μM H_2_DCFDA and 10 μM Hoechst 33342 at 37 °C for 30 min, washed three times with PBS, and imaged on a DMi8 Leica fluorescence microscope (Leica Microsystems, Wetzlar, Germany). ROS fluorescence intensity was analyzed using ImageJ (version 1.46).

### 2.11. Transmission Electron Microscopy (TEM)

Mitochondrial ultrastructures in WT and *Atf4^−/−^* 661W cells treated with 5 μM atRAL for 6 h were examined by TEM, following established protocols [31].

### 2.12. Confocal Fluorescence Imaging

At 37 °C, cells were stained with MitoTracker™ Red CMXRos (10 μM, 30 min) (Cat. No. 40741ES50; Yeasen, Shanghai, China) to visualize mitochondria, Rhodamine-123 (10 μM, 30 min) to evaluate mitochondrial membrane potential (ΔΨm), and MitoSOX™ Red (5 μM, 30 min) (Cat. No. M36008; Thermo Fisher Scientific, Waltham, MA, USA) to detect mitochondrial ROS. Nuclei were labeled with 10 μM Hoechst 33342. After three washes with PBS, fluorescence images were acquired on an Olympus FV4000 confocal microscope (Olympus/Evident, Tokyo, Japan).

### 2.13. Fundus Imaging and Optical Coherence Tomography (OCT)

Mice were anesthetized with 1% pentobarbital sodium (50 mg/kg, i.p.) and received 1% tropicamide eye drops for mydriasis before imaging. Fundus and OCT scans were acquired using the Optoprobe system [32], with subsequent layer thickness measurements; the total retina and outer nuclear layer (ONL) were analyzed via ImageJ (version 1.46).

### 2.14. Electroretinography (ERG)

Full-field ERG was performed as described previously [32]. Mice were dark-adapted overnight, followed by pupillary dilation (1% tropicamide) and anesthesia with isoflurane. Scotopic ERG responses were recorded using an animal ERG system (Diagnosys LLC, Lowell, MA, USA) with white-flash stimuli of increasing intensities (0.01, 0.1, 1, 3, and 10 cd·s/m^2^). a- and b-wave amplitudes were measured. To minimize circadian variation, all recordings were conducted at the same time daily.

### 2.15. Hematoxylin and Eosin (H&E) Staining

Eyes were collected and fixed in FAS eyeball fixative (Servicebio; Wuhan, China) at 4 °C overnight. Following dehydration and paraffin embedding, 5 μm serial sections were prepared through the optic nerve head. H&E staining was performed with a commercial kit, and sections were imaged on a Leica DM2500 microscope (Leica Microsystems, Wetzlar, Germany) [33]. ONL thickness was measured at 16 predefined points per section (±250, ±500, ±750, ±1000, ±1250, ±1500, ±1750, and ±2000 μm from the optic nerve center) using Leica LAS X software (version 3.4.1.17822).

### 2.16. Immunofluorescence Staining

Cells were fixed (15 min), permeabilized with 0.2% Triton X-100 (20 min), and blocked with 2% BSA (1 h) at room temperature. Paraffin sections were dewaxed, antigen-retrieved, and blocked with 2% BSA (1 h). For RPE/choroid flat mounts, eyes were fixed for 30 min before dissecting the RPE/choroid complex, followed by permeabilization (0.2% Triton X-100, 20 min) and blocking (2% BSA/0.2% Triton X-100, 1 h). Samples were incubated with primary antibodies (1:200, overnight, 4 °C) and secondary antibodies (1:400, 2 h, room temperature). Nuclei were stained with DAPI. Images were captured on an Olympus FV4000 confocal microscope (Olympus/Evident, Tokyo, Japan).

### 2.17. Western Blotting

Western blotting was performed as reported [9]. Membranes were incubated with primary rabbit antibodies (1:1000) targeting ATF4, cleaved caspase-3, PARP, γH2AX, GSDME, HMGB1, or GAPDH overnight at 4 °C, then with HRP-conjugated goat anti-rabbit secondary antibody (1:5000 dilution; 2 h, room temperature). Protein signals were visualized by ECL and recorded using a ChemiDoc XRS+ imager (Bio-Rad, Hercules, CA, USA).

### 2.18. Quantitative PCR (qPCR)

Total RNA was extracted from cultured cells with TRIeasy reagent, and RNA quality was assessed using a Nanodrop-2000 (Thermo Fisher Scientific, Waltham, MA, USA). Reverse transcription of 1 μg total RNA was performed with ReverTra Ace qPCR RT Master Mix. qPCR was conducted using Taq Pro Universal SYBR qPCR Master Mix on a LightCycler 96 instrument (Roche, Basel, Switzerland). Gene expression levels were normalized to *Gapdh*. Primer sequences, designed with NCBI Primer Designer, are listed in Supplementary Table S3. All primers were obtained from Sangon Biotech (Shanghai, China).

### 2.19. Statistical Analyses

Values represent mean ± SD of at least three independent experiments, analyzed with GraphPad Prism 8.0 (La Jolla, CA, USA). Statistical significance was evaluated by Student’s *t*-test or ANOVA (one- or two-way) with Tukey’s or Bonferroni’s multiple comparisons, as noted in figure legends. *p* < 0.05 indicated statistical significance.

## 3. Results

### 3.1. atRAL Activates ISR-ATF4 Signaling in 661W Cells

**Figure 1:**
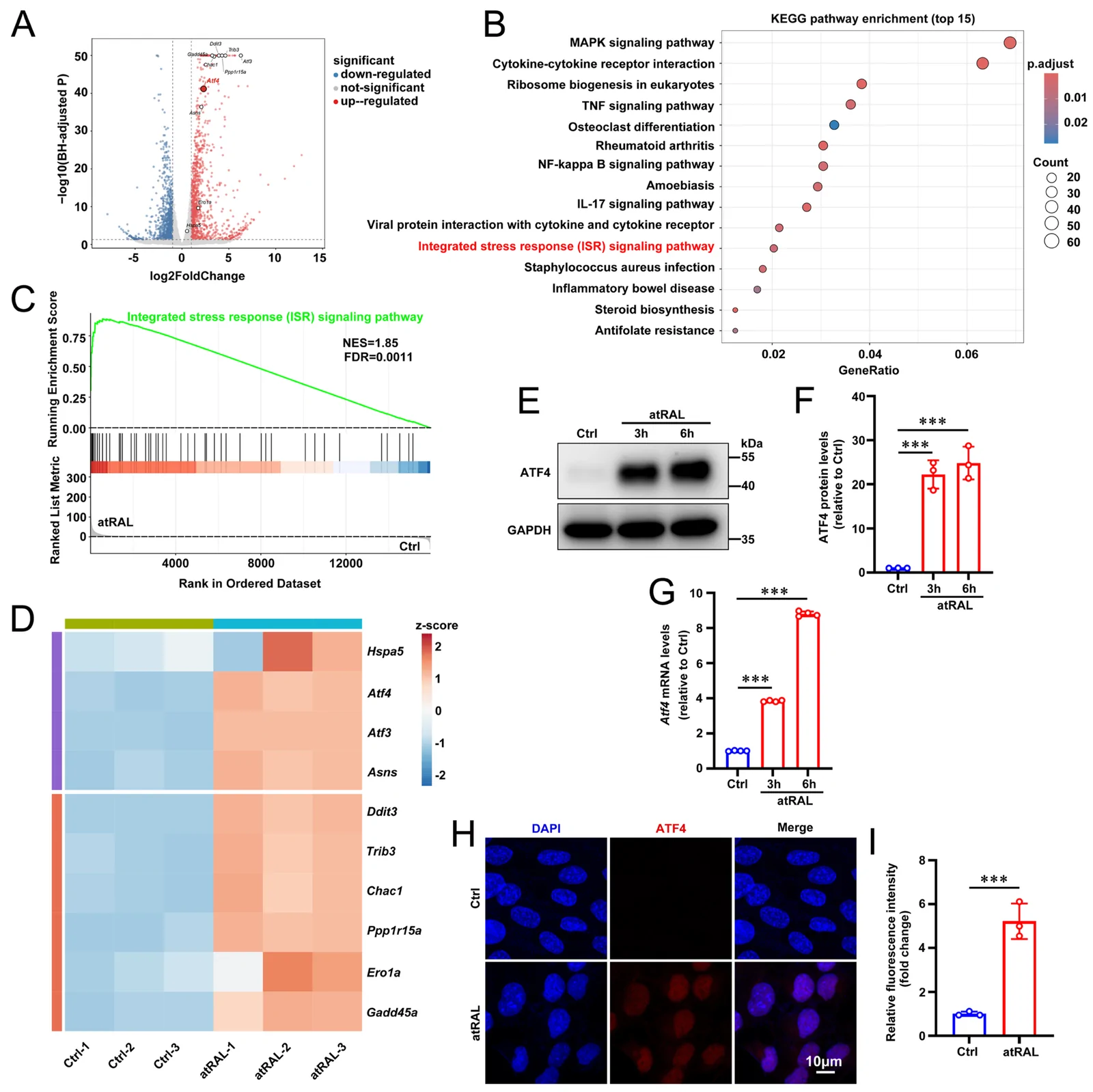
atRAL induces ISR–ATF4-associated transcriptional response in 661W cells. (**A**) Volcano plot of differentially expressed genes in 661W cells treated with 5 μM atRAL or DMSO vehicle for 6 h. Red and blue dots represent upregulated and downregulated genes, respectively (|log_2_FC| ≥ 1, adjusted *p* < 0.05). Selected ISR–ATF4-associated genes are labeled. (**B**) KEGG over-representation analysis of differentially expressed genes following atRAL treatment. Bubble size represents the number of genes assigned to each pathway, and color indicates the Benjamini–Hochberg-adjusted *p* value. The ISR signaling pathway is highlighted. (**C**) GSEA of the ISR signaling pathway. NES, normalized enrichment score; FDR, false discovery rate. (**D**) Heatmap showing the expression of selected genes associated with ISR–ATF4 stress signaling. (**E**,**F**) Western blots and quantification of ATF4 in 661W cells exposed to 5 μM atRAL for 3 and 6 h (*n* = 3). GAPDH was used as a loading control. (**G**) qPCR analysis of *Atf4* mRNA expression in 661W cells treated with 5 µM atRAL for 3 and 6 h (*n* = 4). (**H**,**I**) Immunofluorescence staining and quantification of ATF4 (*Red*) in 661W cells after incubating with 5 μM atRAL for 6 h (*n* = 3). Nuclei were stained with DAPI (*blue*). The scale bars represent 10 μm. Control cells were treated with DMSO alone for 6 h. Statistical analysis was performed using one-way ANOVA followed by Tukey’s multiple comparison test in (**F**,**G**), and by Student’s *t*-test in (**I**). *** *p* < 0.001.

Derived from a murine retinal tumor, 661W cells represent a cone photoreceptor model that is routinely employed to study the pathogenesis of retinal degeneration [34]. Our previous study demonstrated that atRAL increased eIF2α phosphorylation in 661W cells and that suppression of eIF2α signaling attenuated atRAL-induced cell death [35]. We therefore investigated whether the downstream ISR effector ATF4 was activated following atRAL exposure. Reanalysis of a previously generated transcriptomic dataset revealed extensive differential gene expression in atRAL-treated 661W cells compared with vehicle-treated controls (Figure 1A). KEGG over-representation analysis identified the ISR signaling pathway among the significantly enriched pathways (Figure 1B), whereas GSEA revealed marked enrichment of the ISR gene set in the atRAL-treated group (NES = 1.85, FDR = 0.0011) (Figure 1C). Consistent with the pathway-level analyses, focused analysis showed coordinated upregulation of genes associated with endoplasmic reticulum (ER) stress and ATF4 transcriptional activity, including *Hspa5*, *Atf4*, *Atf3*, and *Asns*. Genes associated with sustained ISR-ATF4 stress signaling were also increased, including *Ddit3*, *Trib3*, *Chac1*, *Ppp1r15a*, *Ero1a*, and *Gadd45a* (Figure 1D). These transcriptional changes suggest induction of an ATF4-associated transcriptional program. We next validated ATF4 activation at the mRNA and protein levels. Consistent with this, qPCR and Western blot analyses revealed that treating 661W cells with 5 μM atRAL for 3 and 6 h induced a time-dependent increase in both *Atf4* mRNA and protein levels (Figure 1E–G). Immunofluorescence staining further verified that ATF4 protein was markedly upregulated in 661W cells after 6 h of 5 μM atRAL exposure (Figure 1H,I). Enhanced nuclear accumulation of ATF4 was also observed in atRAL-treated 661W cells (Figure 1H,I). Together, these findings demonstrate that atRAL activates the ISR-ATF4 signaling pathway in 661W cells.

### 3.2. Knockout of the Atf4 Gene Rescues 661W Cells from Apoptosis Induced by atRAL

**Figure 2:**
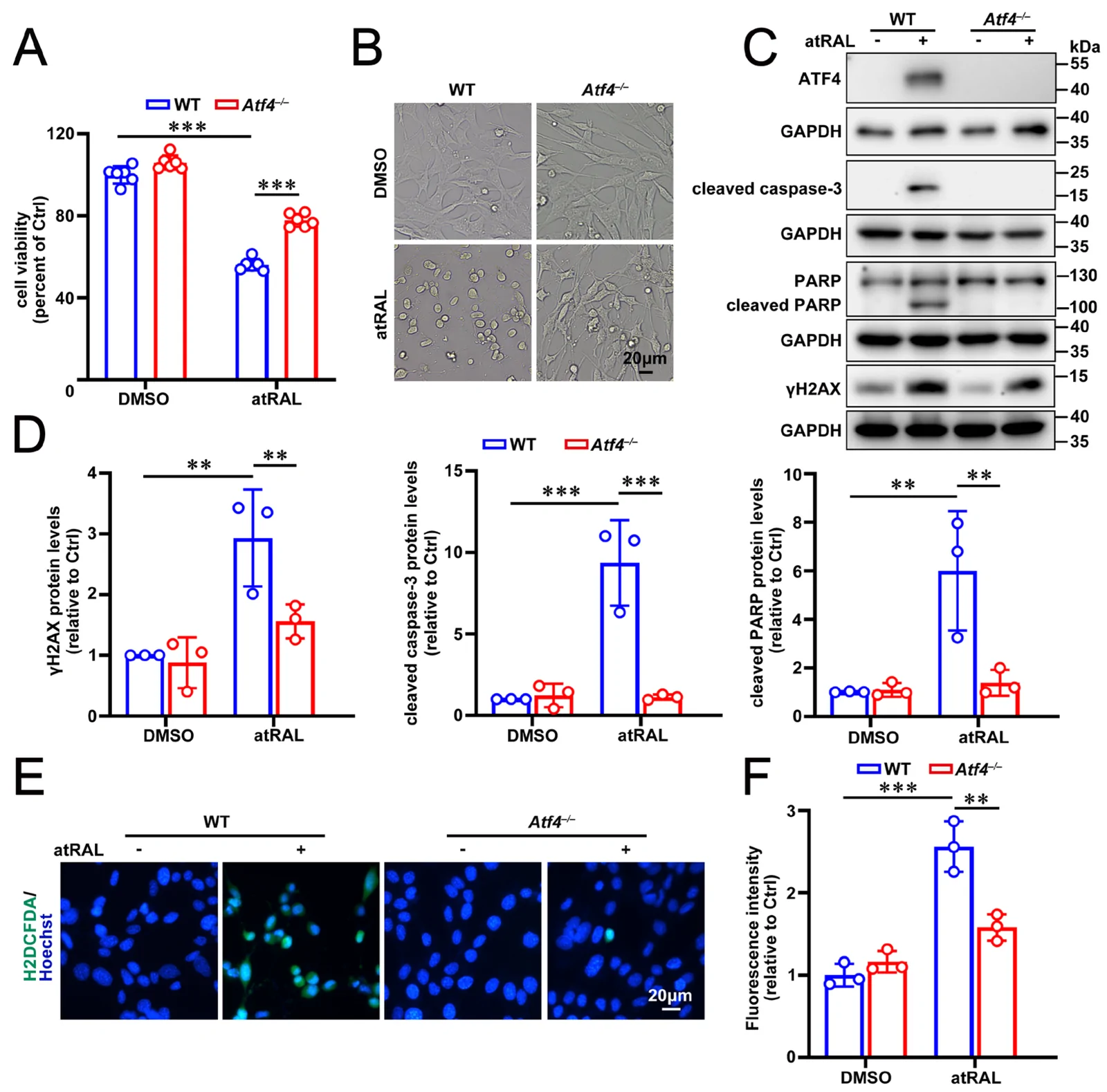
Genetic ablation of *Atf4* preserves the viability and morphology of atRAL-loaded 661W cells by suppressing apoptosis and ROS production. WT and *Atf4^−/−^* 661W cells were treated with 5 μM atRAL or DMSO for 6 h, respectively. (**A**) Cell viability was assessed by MTS assay (*n* = 6). (**B**) Representative images of cellular morphology were captured using a Leica DMi8 inverted microscope. Scale bars, 20 μm. (**C**,**D**) Immunoblots of ATF4, cleaved caspase-3, PARP, cleaved PARP and γH2AX, and quantitative analysis of ATF4, cleaved caspase-3, cleaved PARP and γH2AX (*n* = 3). (**E**,**F**) Intracellular ROS levels were visualized and quantified using green-fluorescent probe H2DCFDA (*n* = 3). Scale bars, 20 μm. Statistical analyses in (**A**,**D**,**F**) were performed using two-way ANOVA with Tukey’s multiple comparison test. ** *p* < 0.01, *** *p* < 0.001.

To further confirm the involvement of ATF4 in atRAL-induced 661W cell death, we established *Atf4*-knockout (*Atf4^−/−^*) 661W cells via the CRISPR-Cas9 system. Ablation of the *Atf4* gene markedly enhanced cell survival after a 6 h challenge with 5 μM atRAL, as assessed by viability assays and corroborated by morphological evaluation (Figure 2A,B). Western blotting verified the complete loss of ATF4 protein in *Atf4^−/−^* 661W cells exposed to 5 μM atRAL for 6 h (Figure 2C). The proteolytic cleavage of caspase-3 and its downstream substrate poly(ADP-ribose) polymerase (PARP), well-established markers of caspase-dependent apoptosis [36,37], was significantly diminished in atRAL-treated *Atf4^−/−^* 661W cells relative to WT controls upon atRAL exposure (Figure 2C,D). These observations demonstrate that ATF4 functions upstream of caspase-3 activation under these conditions. Likewise, levels of γH2AX (phospho-Ser^139^H2AX), an early indicator of DNA double-strand breaks [38], were considerably lower in *Atf4^−/−^* 661W cells after atRAL treatment (Figure 2C,D). This implies that ATF4 suppression effectively attenuates atRAL-induced DNA damage in 661W cells. Furthermore, fluorescence microscopy using the probe 2′,7′-dichlorodihydrofluorescein diacetate (H2DCFDA) demonstrated that *Atf4* deficiency markedly attenuated intracellular ROS production in 661W cells exposed to atRAL (Figure 2E,F). Collectively, these findings reveal that atRAL-induced upregulation of ATF4 promotes photoreceptor apoptosis, at least in part, through caspase activation and ROS production.

### 3.3. Deficiency of the Atf4 Gene Protects atRAL-Loaded 661W Cells from Pyroptosis and Mitochondrial Damage

**Figure 3:**
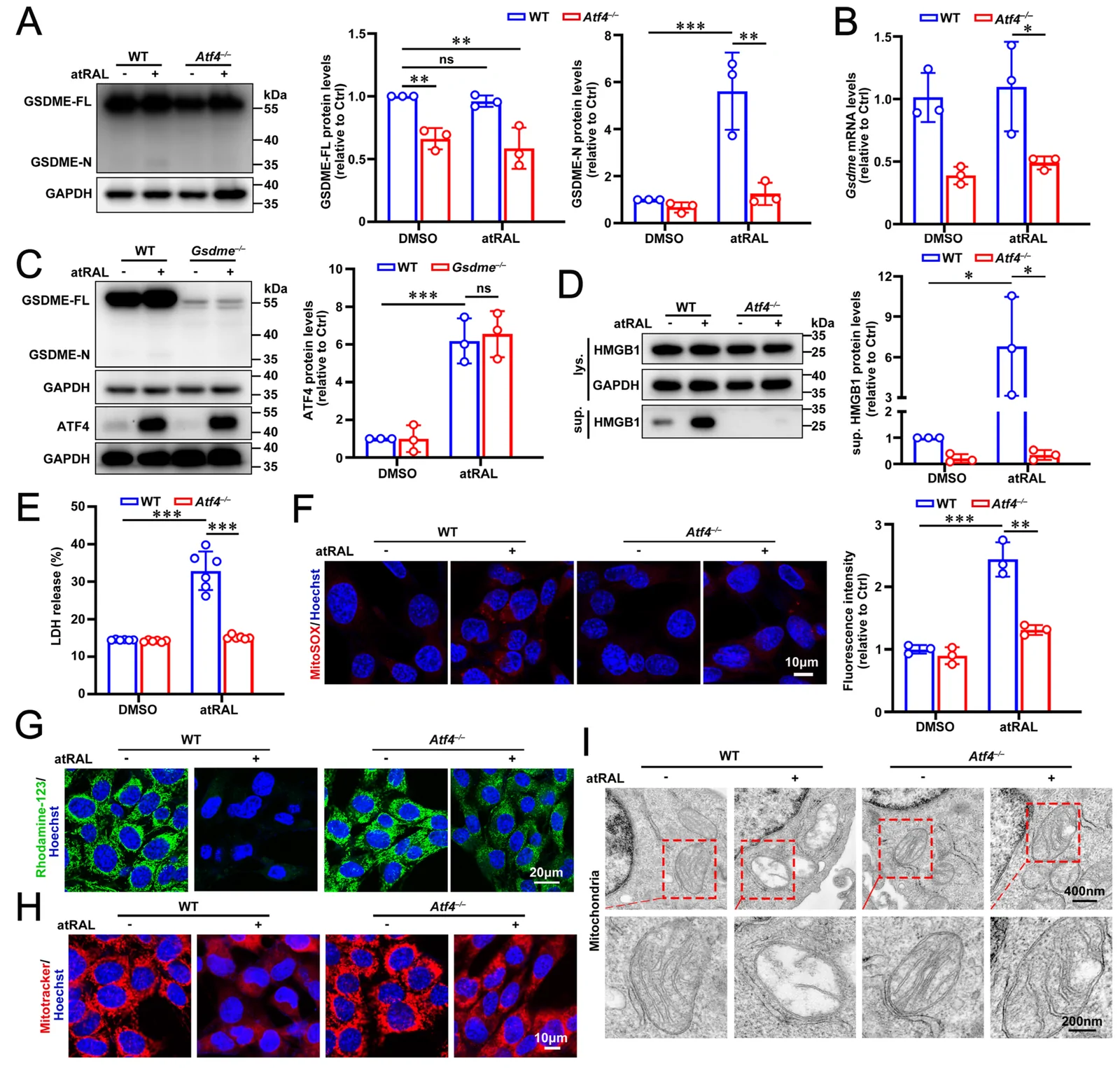
Deficiency of *Atf4* gene protects 661W cells from atRAL-induced pyroptosis and mitochondrial impairment. Cells were treated with 5 μM atRAL or DMSO for 6 h. GAPDH served as an internal loading control. (**A**) Immunoblots and quantitative analysis of GSDME-FL and GSDME-N in WT and *Atf4^−/−^* 661W cells (*n* = 3). (**B**) qPCR analysis of *Gsdme* in WT and *Atf4^−/−^* 661W cells (*n* = 3). (**C**) Western blots of GSDME-FL, GSDME-N and ATF4, and quantification of ATF4 in WT and *Gsdme^−/−^* 661W cells (*n* = 3). (**D**) Immunoblot analysis of HMGB1 in cell lysates and culture supernatants from WT and *Atf4^−/−^* 661W cells (*n* = 3). (**E**) Plasma membrane rupture was evaluated by LDH release assay (*n* = 3). (**F**) Mitochondrial superoxide production was assessed using MitoSOX^TM^ Red staining (red) (*n* = 3). Scale bars, 10 μm. (**G**) ΔΨm was measured by Rhodamine-123 staining (Green). Scale bars, 20 μm. (**H**) The morphology of mitochondria was visualized using MitoTracker Red CMXRos staining (red). Scale bars, 10 μm. (**I**) Typical TEM images showing mitochondrial ultrastructures in WT and *Atf4^−/−^* 661W cells. Scale bars, 400 nm (upper panels) and 200 nm (lower panels). Statistical analyses in (**A**–**F**) were performed using two-way ANOVA with Tukey’s multiple comparison test. * *p* < 0.05, ** *p* < 0.01, *** *p* < 0.001. ns, not significant.

Apoptosis and GSDME-mediated pyroptosis converge on a common upstream regulator, caspase-3 [39]. High GSDME expression combined with caspase-3 activation is required for the switch from apoptosis to pyroptosis [39]. Based on this, ATF4 may participate in the transcriptional control of GSDME in 661W cells. To address this possibility, we assessed GSDME expression in 661W cells following genetic ablation of *Atf4*. Both mRNA and protein levels of GSDME were substantially decreased in *Atf4^−/−^* 661W cells under basal conditions and after exposure to 5 μM atRAL (Figure 3A,B). Moreover, protein levels of the *N*-terminal of GSDME (GSDME-N), the executor of pyroptosis, reflected the extent of GSDME cleavage, and were significantly diminished in atRAL-loaded *Atf4^−/−^* compared to WT 661W cells, but remained intact under basal conditions (Figure 3A). Conversely, ATF4 protein levels were unaltered in *Gsdme^−/−^* versus WT 661W cells after atRAL exposure (Figure 3C), indicating that ATF4 functions upstream of GSDME and likely regulates its transcription. ATF4 deficiency strongly decreased HMGB1 release into culture supernatants of 661W cells in response to atRAL (Figure 3D). Likewise, LDH release assays demonstrated that *Atf4* gene knockout preserved plasma membrane integrity and significantly prevented LDH release in atRAL-treated 661W cells (Figure 3E). These two lines of evidence reveal a role of ATF4 in promoting pyroptotic membrane rupture. GSDME-N has been reported to localize to mitochondria, form pores in the mitochondrial membrane, trigger mitochondrial damage, and promote release of cytochrome *c* (Cyt *c*), which in turn activates caspase-9 and caspase-3 [26]. Given this, we next investigated the impact of *Atf4* deficiency on the mitochondria in 661W cells exposed to atRAL. Genetic ablation of *Atf4* markedly lowered mitochondrial superoxide production and significantly attenuated the loss of mitochondrial membrane potential (ΔΨm), as revealed by MitoSOX Red staining and Rhodamine-123 staining, respectively (Figure 3F,G). Confocal imaging with MitoTracker Red CMXRos staining revealed that mitochondrial aggregation induced by atRAL was visibly alleviated in *Atf4^−/−^* 661W cells (Figure 3H). Ultrastructural analysis by TEM showed severe mitochondrial disruption in atRAL-treated WT cells, whereas mitochondrial ultrastructure remained largely preserved in *Atf4^−/−^* 661W cells after exposure to atRAL (Figure 3I). Taken together, these data imply that ATF4 activation promotes GSDME-mediated pyroptosis and mitochondrial damage.

### 3.4. ATF4 Inhibitor ISRIB Promotes the Survival of 661W Cells in Response to atRAL by Repressing Apoptosis, Pyroptosis, ROS Production and Mitochondrial Impairment

**Figure 4:**
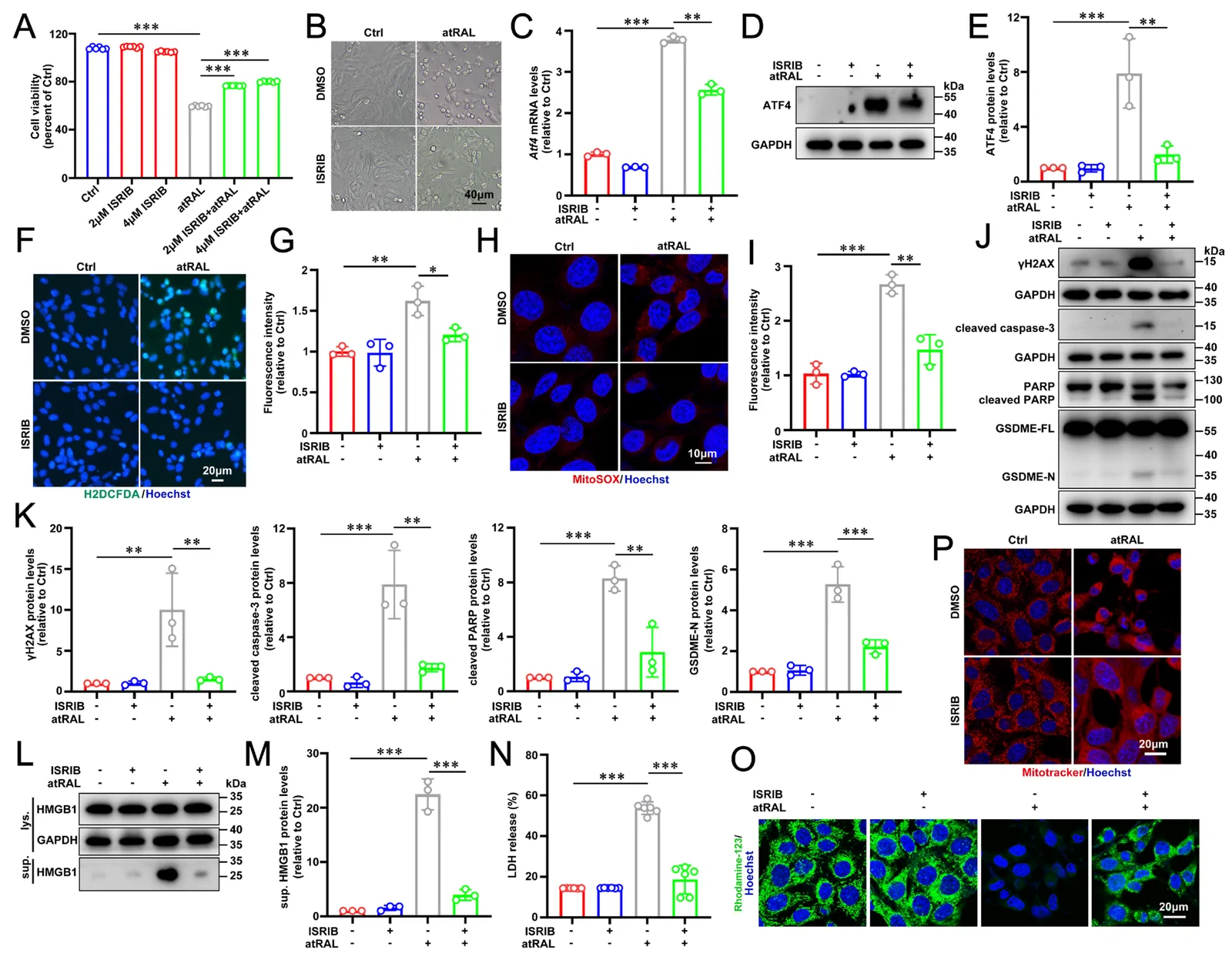
Treatment with ISRIB, an inhibitor of ATF4, promotes the survival of 661W cells in response to atRAL by attenuating pyroptotic cell death and mitochondrial damage. (**A**) Cell viability was assessed by MTS assay (*n* = 6). 661W cells were pretreated with ISRIB at gradient concentrations of 0, 2 and 4 μM for 2 h and then exposed to 5 μM atRAL for 6 h. (**B**–**P**) 661W cells were pretreated for 2 h with 2 μM ISRIB and then incubated with 5 μM atRAL for 6 h. (**B**) Cellular morphology was imaged by the Leica DMi8 inverted microscope. Scale bars, 40 μm. (**C**) The mRNA levels of *Atf4* were analyzed by qPCR (*n* = 3). (**D**,**E**) Western blots and quantification of ATF4 (*n* = 3). (**F**,**G**) Intracellular ROS production was analyzed using the fluorescent probe H2DCFDA by fluorescence microscopy (*n* = 3). Scale bars, 20 μm. (**H**,**I**) The levels of mitochondrial ROS were examined by MitoSOX^TM^ Red staining (*n* = 3). Scale bars, 10 μm. (**J**,**K**) Immunoblots of γH2AX, cleaved caspase-3, PARP, cleaved PARP, GSDME-FL and GSDME-N, and quantification of γH2AX, cleaved caspase-3, cleaved PARP and GSDME-N (*n* = 3). (**L**,**M**) Immunoblots of HMGB1 in cell lysates or culture supernatants, and quantification of HMGB1 in culture supernatants (*n* = 3). (**N**) Rupture of the plasma membrane was assessed by LDH release assay (*n* = 6). (**O**) ΔΨm was assessed by Rhodamine-123 staining. Scale bars, 20 μm. (**P**) Mitochondrial morphology was examined by MitoTracker Red CMXRos staining. Scale bars, 20 μm. Statistical analyses in (**A**,**C**,**E**,**G**,**I**,**K**,**M**,**N**) were performed using one-way ANOVA with Tukey’s multiple comparison test. * *p* < 0.05, ** *p* < 0.01, *** *p* < 0.001.

A small molecule termed N, N′-*trans*-(cyclohexane-1,4-diyl)-bis-(2-(4-chlorophenoxy) acetamide (ISRIB) has recently been shown to strongly reduce ATF4 expression and function as a selective and potent inhibitor of the ATF4 signaling [15,19]. ISRIB allosterically antagonizes phosphorylated eIF2α (p-eIF2α) binding to eIF2B, thereby restoring eIF2B guanine-nucleotide exchange activity and counteracting ISR-dependent translational repression and ATF4 induction [11,40]. MTS assays demonstrated that 2 and 4 μM ISRIB markedly increased the survival of 661W cells following 6 h treatment with 5 μM atRAL (Figure 4A). Treatment with 2 μM ISRIB substantially preserved the morphology of 661W cells following exposure to atRAL (Figure 4B), and it suppressed ATF4 induction by atRAL at mRNA and protein levels (Figure 4C–E). In addition, ISRIB substantially suppressed intracellular ROS and mtROS generation in atRAL-treated 661W cells (Figure 4F–I). ISRIB noticeably diminished pro-apoptotic signaling in 661W cells following atRAL exposure, evidenced by decreased cleaved caspase-3, cleaved PARP, and γH2AX (Figure 4J,K). Similarly, treatment with ISRIB significantly decreased GSDME-N formation in atRAL-loaded 661W cells, and strongly reduced the release of HMGB1 and LDH into culture supernatants (Figure 4J–N), reflecting the ability of ISRIB to relieve pyroptotic cell death. Furthermore, ISRIB markedly attenuated the loss of ΔΨm and substantially suppressed mitochondrial aggregation in atRAL-treated 661W cells (Figure 4O,P). In aggregate, these results indicate that ISRIB confers resistance to atRAL-mediated cell death in 661W cells by attenuating ATF4 signaling, thereby limiting caspase-dependent apoptosis, GSDME-mediated pyroptosis, and mitochondrial impairment.

### 3.5. Inhibition of ATF4 Activation Ameliorates Light-Induced Retinal Damage in Abca4^−/−^Rdh8^−/−^ Mice

**Figure 5:**
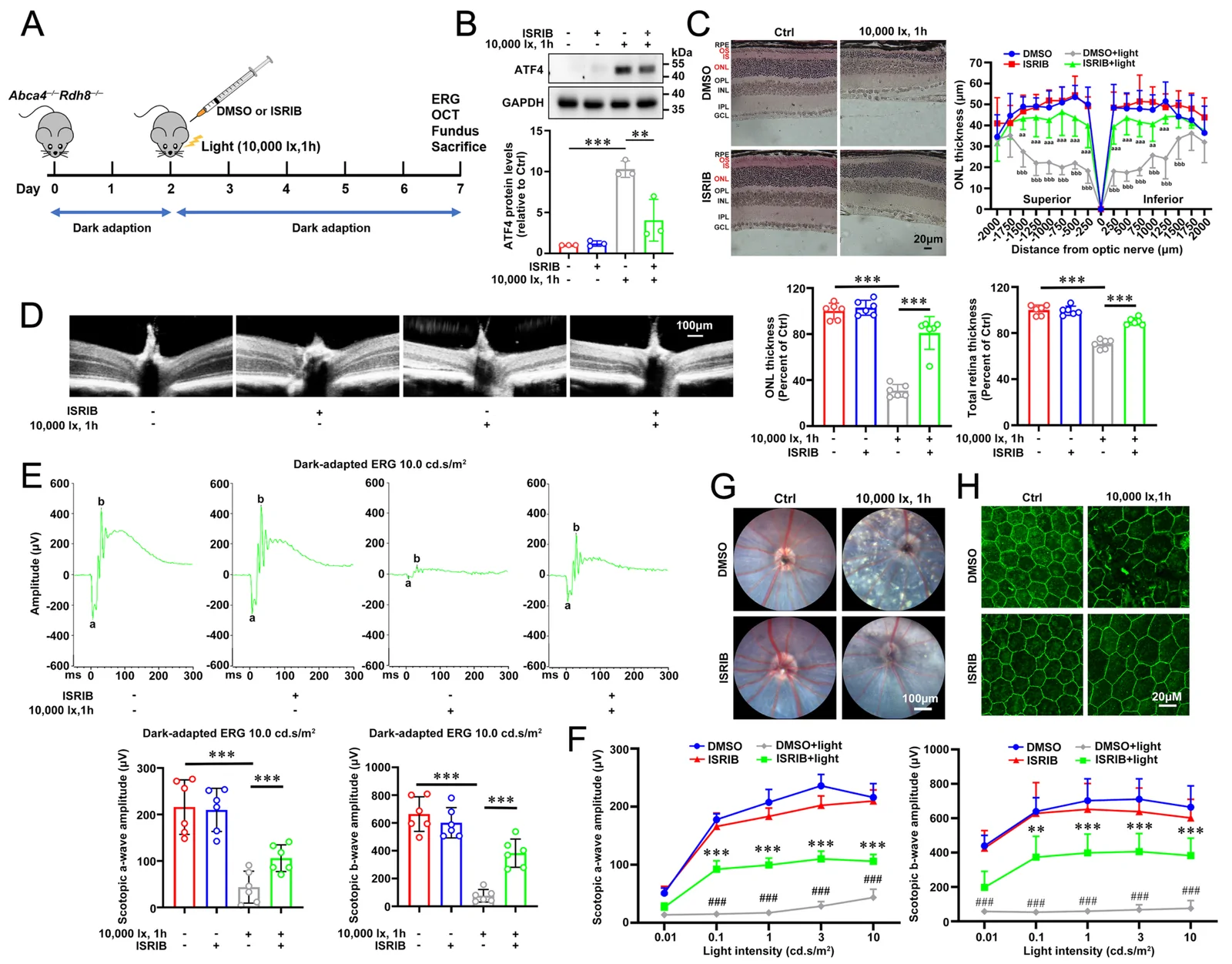
Intraperitoneally injected ATF4 inhibitor ISRIB ameliorates retinal degeneration in light-exposed *Abca4^−/−^Rdh8*^−/−^ mice. (**A**) Schematic illustration of the experimental workflow. (**B**) Western blots and quantification of ATF4 in mouse neural retina (*n* = 3). ** *p* < 0.01, *** *p* < 0.001 by one-way ANOVA with Tukey’s multiple comparison test. (**C**) Histological analysis of mouse retina was performed using H&E staining. RPE, retinal pigment epithelium; OS, outer segment; IS, inner segment; ONL, outer nuclear layer; OPL, outer plexiform layer; INL, inner nuclear layer; IPL, inner plexiform layer; GCL, ganglion cell layer. Scale bars, 20 μm. ONL thickness measurement on the Superior–Inferior meridian (*n* = 6). Two-way ANOVA with Bonferroni’s multiple comparison test. ^aa^ *p* < 0.01, ^aaa^ *p* < 0.001 versus DMSO-treated, light-exposed *Abca4^−/−^Rdh8^−/−^* mice. ^bbb^ *p* < 0.001 versus DMSO-treated, light-free *Abca4^−/−^Rdh8^−/−^* mice. (**D**) OCT imaging. The thickness of the ONL and total retina was quantified at a distance of 400 μm from the optic nerve (*n* = 6). Scale bars, 100 μm. *** *p* < 0.001 by one-way ANOVA with Tukey’s multiple comparison test. (**E**) Scotopic ERG, 10 cd s/m^2^ (*n* = 6). *** *p* < 0.001 by one-way ANOVA with Tukey’s multiple comparison test. (**F**) Scotopic ERG with gradient stimulus luminance of 0.01, 0.1, 1, 3 and 10 cd s/m^2^ (*n* = 6). ** *p* < 0.01, *** *p* < 0.001 versus the DMSO-treated, light-exposed *Abca4^−/−^Rdh8^−/−^* mice; ### *p* < 0.001 versus DMSO-treated, light-free *Abca4^−/−^Rdh8^−/−^* mice. Two-way ANOVA with Bonferroni’s multiple comparison test. (**G**) Fundus photography. Scale bars, 100 μm. (**H**) ZO-1 immunostaining. Scale bars, 20 μm.

Dark-adapted *Abca4^−/−^Rdh8^−/−^* mice received intraperitoneal ISRIB (5 mg/kg) or vehicle (DMSO) 2 h prior to pupil dilation and subsequent 1 h LED light exposure, with daily ISRIB or DMSO injections thereafter for four additional days, as schematically illustrated in Figure 5A. As anticipated, ISRIB treatment led to a significant downregulation of ATF4 expression in the neural retina of light-challenged *Abca4^−/−^Rdh8^−/−^* mice (Figure 5B). OCT imaging and H&E analyses showed that intraperitoneal ISRIB administration substantially attenuated neural retinal injury and prevented the reduction in ONL and total retinal thickness in light-exposed *Abca4^−/−^Rdh8^−/−^* mice (Figure 5C,D). Dark-adapted full-field ERG revealed that ISRIB preserved a- and b-wave amplitudes that were otherwise reduced in *Abca4^−/−^Rdh8^−/−^* mice by light exposure, indicating the rescue of retinal function (Figure 5E,F). Fundus imaging demonstrated that ISRIB treatment alleviated the punctate lesions in the fundus of *Abca4^−/−^Rdh8^−/−^* mice upon light exposure (Figure 5G). Moreover, ZO-1 immunostaining showed that intraperitoneally injected ISRIB preserved the RPE tight junctions in *Abca4^−/−^Rdh8^−/−^* mice following exposure to light (Figure 5H). Collectively, these data indicate that repressing ATF4 expression effectively ameliorates light-induced retinal structural alterations and rescues retinal function in *Abca4^−/−^Rdh8^−/−^* mice.

### 3.6. Repressing ATF4 Signaling Relieves Photoreceptor Apoptosis and Pyroptosis in Light-Challenged Abca4^−/−^Rdh8^−/−^ Mice

**Figure 6:**
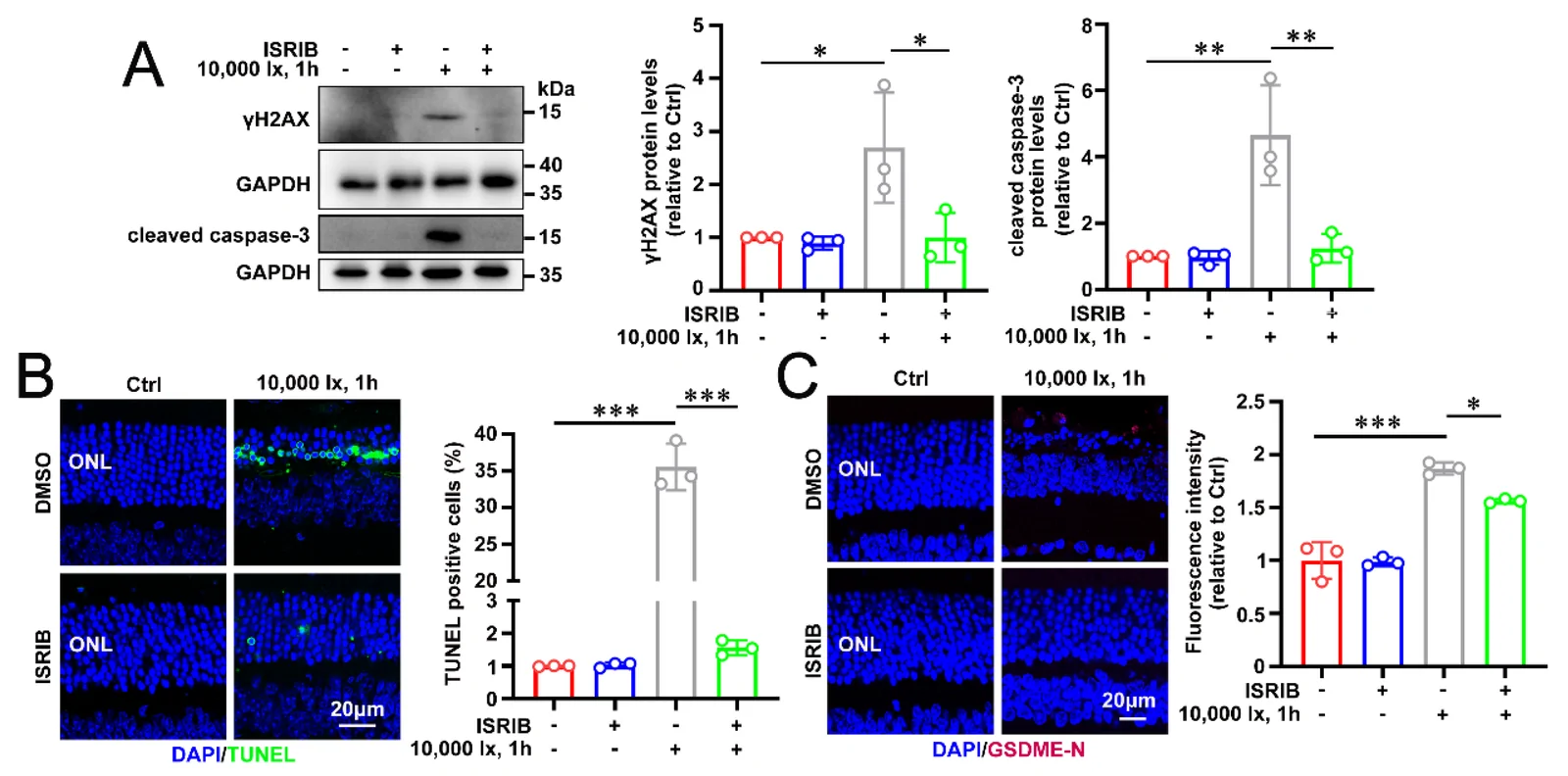
Intraperitoneal administration of ATF4 repressor ISRIB relieves light-induced photoreceptor apoptosis and pyroptosis in *Abca4^−/−^Rdh8*^−/−^ mice. (**A**) Immunoblots and quantification of γH2AX and cleaved caspase-3 in mouse neural retina (*n* = 3). (**B**) Apoptotic photoreceptor cells were examined by TUNEL staining (*n* = 3). (**C**) Immunofluorescence analysis and quantification of GSDME-N (*n* = 3). * *p* < 0.05, ** *p* < 0.01, *** *p* < 0.001 by one-way ANOVA with Tukey’s multiple comparison test. Scale bars in B and C, 20 μm.

To determine the mechanisms behind the in vivo benefits of inhibiting ATF4 with ISRIB, we analyzed markers for apoptosis and GSDME-mediated pyroptosis in the neural retina of light-stressed *Abca4^−/−^Rdh8^−/−^* mice. Consistent with our cellular findings, ISRIB treatment led to a marked downregulation of cleaved caspase-3 and γH2AX protein levels within the neural retina of light-challenged *Abca4^−/−^Rdh8^−/−^* mice (Figure 6A). TUNEL staining demonstrated that ISRIB significantly reduced light-induced photoreceptor apoptosis in light-stressed *Abca4*^−/−^*Rdh8*^−/−^ mice (Figure 6B). Immunofluorescence analysis further revealed diminished GSDME-N immunoreactivity in photoreceptors of light-challenged *Abca4^−/−^Rdh8^−/−^* mice upon ISRIB treatment (Figure 6C). Overall, these results indicate that ATF4-dependent activation of both apoptosis and GSDME-mediated pyroptosis contributes to light-triggered retinal atrophy in *Abca4*^−/−^*Rdh8*^−/−^ mice, and that pharmacological inhibition of the ATF4/GSDME axis by ISRIB attenuates these detrimental processes and helps maintain retinal integrity.

## 4. Discussion

Dry AMD and STGD1 are characterized by progressive degeneration of photoreceptors and the RPE in the macula, ultimately leading to central vision loss. The molecular pathways linking visual-cycle dysfunction to photoreceptor demise remain incompletely understood. Our data identified sustained ISR–ATF4 signaling as a critical mediator of atRAL-induced retinal injury. atRAL exposure induced an ISR-ATF4-associated transcriptional program, increased ATF4 abundance and nuclear accumulation, and promoted both photoreceptor apoptosis and GSDME-mediated pyroptosis, whereas genetic deletion or pharmacological inhibition of ATF4 conferred photoreceptor and RPE protection (Figure 1, Figure 2, Figure 3, Figure 4, Figure 5 and Figure 6). Collectively, these findings identify ATF4 as a novel pathogenic driver that may contribute to retinal degeneration in dry AMD and STGD1.

The ISR and its key effector, ATF4, play context-dependent roles in maintaining cellular homeostasis [18]. Under conditions of low-grade stress, transient activation of ATF4 induces adaptive programs, such as the expression of chaperones and antioxidant genes, which work to restore proteostasis and metabolic balance [41]. In contrast, sustained ATF4 signaling promotes maladaptive remodeling, metabolic imbalance, and ultimately cellular apoptosis [17,18,42]. Within the retina, ATF4 has been reported to exert dual effects: its downregulation increased the susceptibility of ARPE-19/HPV-16 cells to oxidative stress [43], while its genetic or pharmacological inhibition has been shown to attenuate pathological neovascularization and retinal damage in other experimental animal models [44,45,46].

In this study, we demonstrated the detrimental effect of ATF4 on atRAL-induced retinal degeneration, and further indicated that suppressing ATF4 in photoreceptors mitigated atRAL-triggered oxidative stress, apoptosis, and pyroptosis. It should be noted, however, that germline *Atf4* knockout mice exhibit systemic abnormalities, such as reduced bone mass and microphthalmia [47,48,49]. The observed microphthalmia in these models has been linked to lens degeneration caused by increased lens cell apoptosis, a defect that appears largely confined to the lens, with no major structural anomalies reported in the retina or cornea [50]. Thus, future investigations using retina-specific conditional knockout models are necessary to elucidate the function of ATF4 in adult retinal homeostasis and disease pathogenesis.

Although previous studies have implicated p53, STAT3, and Sp1 in the modulation of GSDME in some biological contexts [27,28,51], the transcriptional regulation of GSDME in photoreceptors has not been defined. Here, we demonstrated that genetic ablation of *Atf4* significantly reduced both *Gsdme* mRNA and protein levels in 661W cells, whereas knockout of *Gsdme* did not affect ATF4 expression (Figure 3A–C). These findings support a unidirectional regulatory relationship in which ATF4 functions as an upstream transcriptional regulator of GSDME. Moreover, deleting *Atf4* significantly diminished GSDME-N protein abundance in 661W cells incubated with atRAL (Figure 3A), and it visibly limited pore formation at the plasma membrane, as revealed by diminished efflux of HMGB1 and LDH (Figure 3D,E). Interestingly, pharmacological inhibition of ATF4 using ISRIB in atRAL-loaded 661W cells clearly reduced ATF4 expression and GSDME-N formation without significantly altering GSDME protein levels (Figure 4D,E,J,K). This discrepancy may reflect that the mechanistic basis underlying ISRIB-mediated reduction of GSDME-N protein levels differs from that of ATF4 knockout in 661W cells exposed to atRAL. In addition, our findings also suggest that exon 3 of ATF4 likely participates in the regulation of GSDME transcription (Supplementary Table S2). Moreover, both knockout and pharmacologic inhibition of ATF4 markedly attenuated 661W cell apoptosis induced by atRAL (Figure 2C,D and Figure 4J,K). More importantly, repression of ATF4 in the neural retina by intraperitoneal administration of ISRIB significantly alleviated caspase-dependent apoptosis and GSDME-dependent pyroptosis as well as reduced the generation of GSDME-N in photoreceptors of light-exposed *Abca4^−/−^Rdh8^−/−^* mice, and it visibly prevented retinal structural and functional damage (Figure 5 and Figure 6). Our previous report has provided strong evidence that activation of GSDME promotes apoptosis in 661W cells treated with atRAL and in neural retina from *Abca4^−/−^Rdh8^−/−^* mice after exposure to light [26], suggesting that GSDME-mediated apoptosis is a partial cause of photoreceptor death resulting from ATF4 activation by atRAL. Taken together, these data indicate that ATF4 activation stimulates the formation of GSDME-N, and triggers caspase-dependent photoreceptor apoptosis and GSDME-dependent photoreceptor pyroptosis and apoptosis.

Following its generation in atRAL-exposed 661W cells, GSDME-N localizes to mitochondria and inserts into the outer membrane to create pores, resulting in mitochondrial damage and Cyt c release that drive caspase-9 and caspase-3 activation [26]. In addition, early studies have demonstrated that accumulation of intracellular ROS, including mitochondrial ROS, and loss of ΔΨm are closely associated with the death of 661W cells following atRAL exposure [9,52]. Furthermore, pharmacologically repressing mitochondrial ROS effectively alleviates retinal functional and structural impairment in light-exposed *Abca4^−/−^Rdh8^−/−^* mice [52]. In the current study, genetic deletion of ATF4 or pharmacologic inhibition of ATF4 with ISRIB reduced production of intracellular ROS, including mitochondrial ROS, preserved ΔΨm and mitochondrial ultrastructure, and attenuated both pyroptosis and apoptosis in atRAL-exposed 661W cells (Figure 2, Figure 3 and Figure 4), implying that atRAL-induced mitochondrial damage, at least in part, drives 661W cell death through ATF4/GSDME axis activation.

This study has several limitations. atRAL-loaded 661W cells and light-exposed *Abca4^−/^^−^Rdh8^−/^^−^* mice are useful models of photoreceptor stress and impaired atRAL clearance, respectively, and recapitulate several key features of STGD1 and dry AMD; nevertheless, neither fully captures the complex and progressive nature of these human retinal diseases. In addition, ISRIB acts on the ISR rather than serving as a specific ATF4 inhibitor; therefore, potential ATF4-independent effects cannot be excluded. The upstream mechanisms underlying sustained ATF4 activation, as well as the relative contributions of apoptosis and GSDME-mediated pyroptosis to retinal degeneration, also warrant further investigation. Finally, the long-term efficacy, ocular safety, optimal dosing regimen, and therapeutic window of ATF4-targeted interventions remain to be established in chronic disease models and human-relevant retinal systems before their translational potential can be fully evaluated.

**Figure 7:**
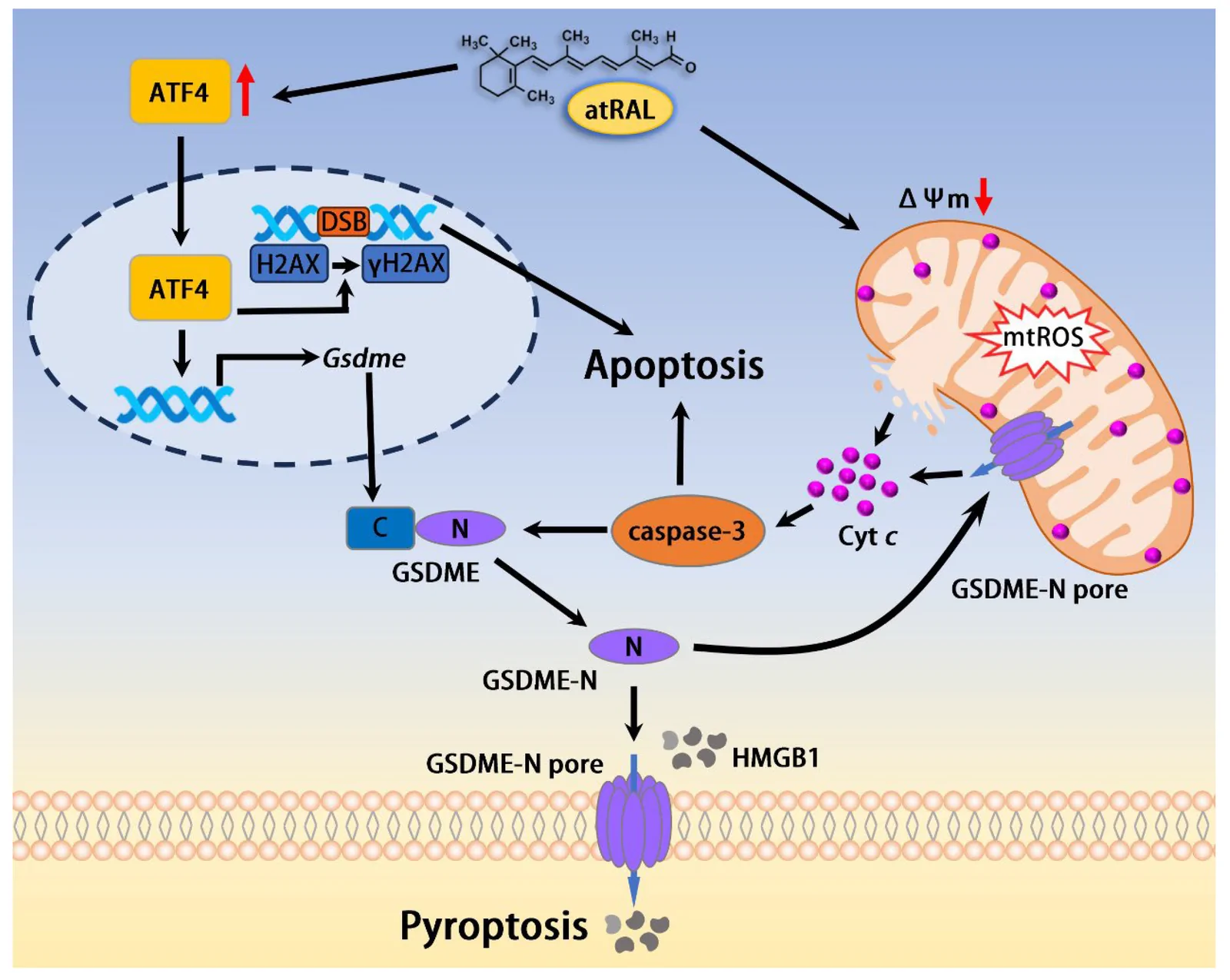
Proposed mechanisms by which the activation of ATF4 by atRAL induces photoreceptor apoptosis and pyroptosis.

Based on the foregoing results, we propose a model in which atRAL-triggered ATF4 activation provokes both apoptosis and pyroptosis in photoreceptors. As depicted in Figure 7, elevated levels of this aldehyde in photoreceptors induce overexpression of the transcription factor ATF4. Once activated, ATF4 translocates to the nucleus where it upregulates *Gsdme* expression and phosphorylates histone H2AX to generate γH2AX, a sensitive marker for DNA double-strand breaks (DSBs). Concurrently, atRAL stimulates mitochondrial ROS production, reduces ΔΨm, and compromises mitochondrial outer membrane integrity, culminating in Cyt c release into the cytosol and subsequent caspase-3 activation. Active caspase-3 and DNA damage jointly trigger apoptosis. In a parallel pathway, caspase-3 cleaves GSDME to produce GSDME-N, which oligomerizes and inserts into the plasma membrane to form pores, thereby executing pyroptosis. Plasma membrane rupture during pyroptosis leads to the release of intracellular contents such as HMGB1. Furthermore, GSDME-N translocates to mitochondria, where it oligomerizes and increases mitochondrial outer membrane permeability, which amplifies both pyroptosis and apoptosis by enhancing Cyt c release and caspase-3 activation. In summary, our study demonstrates that ATF4 drives retinal degeneration in atRAL-related retinopathies through caspase- and DNA damage-dependent apoptosis as well as GSDME-mediated pyroptosis and apoptosis, underscoring ATF4 modulation as a promising translational strategy for STGD1 and dry AMD.

## 5. Conclusions

In summary, the available evidence identifies ATF4 as a critical driver of atRAL-induced retinal atrophy and an upstream transcriptional regulator of GSDME. Notably, both genetic deletion and pharmacological inhibition of ATF4 confer robust retinal protection, underscoring the translational potential of ATF4-targeted therapies for STGD1 and dry AMD.

## Data Availability

All data supporting the conclusions of this study are available in the manuscript and Supplementary Files, or from the corresponding author upon reasonable request.

## Supplementary Materials

The following supporting information can be downloaded at: https://www.mdpi.com/article/10.3390/antiox15101328/s1, Table S1: Detailed reagents and antibodies; Table S2: Guide RNA sequences; Table S3: Primer sequences.
