# Supplementary material for: ATF4 Activation Promotes All-trans-Retinal-Driven Retinopathies in Mice
Source: Antioxidants (Basel). 2026 Oct 14;15(10):1328. doi: 10.3390/antiox15101328 (PMC13701645; doi:10.3390/antiox15101328)
Supplement: Supplementary file 1 [file antioxidants-15-01328-s001.zip › antioxidants-4540365-supplementary.pdf]

Supplementary Materials for

ATF4 Activation Promotes All-*trans*-Retinal-Driven Retinopathies in Mice

Kunhuan Yang [1,2], Chaofeng Yu [2], Danxue He [1,2], Lei Tao [1,2], Bo Yang [1,2], Yuling Chen [1,2], Jingmeng Chen [3] and Yalin Wu [1,2,4,*]

[1] Xiamen Eye Center and Eye Institute of Xiamen University, School of Medicine, Xiamen University, Xiamen 361003, China
[2] Fujian Provincial Key Laboratory of Ophthalmology and Visual Science, Fujian Engineering and Research Center of Eye Regenerative Medicine, Eye Institute of Xiamen University, School of Medicine, Xiamen University, Xiamen 361102, China
[3] School of Medicine, Xiamen University, Xiamen 361102, China
[4] Shenzhen Research Institute of Xiamen University, Shenzhen 518057, China
* Correspondence: yalinw@xmu.edu.cn

This file includes:

Supplementary Table S1 to Table S3.

Supplementary Table S1. Detailed reagents and antibodies.

Reagents/Antibodies	Catalog	Company
All-*trans*-retinal	R2500	Sigma-Aldrich
4',6-diamidino-2-phenylindole (DAPI)	F6057	Sigma-Aldrich
Hoechst 33342	B2261	Sigma-Aldrich
ISRIB	HY-12495A	MedChemExpress
Dimethyl sulfoxide (DMSO)	D8371	Solarbio
2′,7′-dichlorodihydrofluorescein diacetate (H2DCFDA)	D399	Thermo Fisher Scientific
Rhodamine 123	R302	Thermo Fisher Scientific
Mouse monoclonal anti-ZO-1	MA3-39100-A555	Thermo Fisher Scientific
MitoSOX™ Red Mitochondrial Superoxide Indicator	M36008	Thermo Fisher Scientific
TRIeasy total RNA extraction reagent	10606ES60	Yeasen
MitoTracker® Red CMXRos	40741ES50	Yeasen
Rabbit monoclonal anti-ATF4	11815S	Cell Signaling Technology
Rabbit monoclonal anti-cleaved caspase-3	9664S	Cell Signaling Technology
Rabbit polyclonal anti-PARP	9542S	Cell Signaling Technology
Rabbit polyclonal anti-HMGB1	3935S	Cell Signaling Technology
Rabbit monoclonal anti-GSDME	ab222407	Abcam
Rabbit polyclonal anti-γH2AX	NB100-384	Novus
Mouse monoclonal anti-GAPDH (HRP conjugated)	HRP-60004	Proteintech
Hematoxylin and eosin (H&E) staining kit	P032IH	Auragene Biotech
Goat anti-rabbit IgG (H+L) secondary antibody	31460	Thermo Fisher Scientific
Alexa Fluor™ 488-conjugated donkey anti-mouse IgG (H+L) highly cross-adsorbed secondary antibody	A21206	Thermo Fisher Scientific
Alexa Fluor™ 594-conjugated donkey anti-rabbit IgG (H+L) highly cross-adsorbed secondary antibody	A21206	Thermo Fisher Scientific
ReverTra Ace qPCR RT Master Mix kit	FSQ201	Toyobo
Taq Pro Universal SYBR qPCR Master Mix	Q71202	Vazyme

Supplementary Table S2. Guide RNA sequences.

Gene	Forward primer (5′→3′)	Reverse primer (5′→3′)
Atf4	CACCGCCCAAACCCGACTGGTCGAA	AAACTTCGACCAGTCGGGTTTGGGC

Supplementary Table S3. Primer sequences.

Gene	Species	Forward primer (5′→3′)	Reverse primer (5′→3′)
Atf4	mouse	CCTATAAAGGCTTGCGGCCA	GTCCGTTACAGCAACACTGC
Gsdme	mouse	CCACAGTTTTCCTGGGGACT	GCAGAGGCAAACAATCGCTG
Gapdh	mouse	AGGTCGGTGTGAACGGATTTG	TGTAGACCATGTAGTTGAGGTCA
